# Bridging the Gap: Supplements Strategies from Experimental Research to Clinical Applications in Sarcopenic Obesity

**DOI:** 10.3390/cimb46120800

**Published:** 2024-11-24

**Authors:** Bogdana Virgolici, Maria-Zinaida Dobre, Daciana Costina Andrada Stefan

**Affiliations:** Department of Biochemistry, Carol Davila University of Medicine and Pharmacy, 050474 Bucharest, Romania; bogdana.virgolici@umfcd.ro (B.V.); daciana.stefan@umfcd.ro (D.C.A.S.)

**Keywords:** sarcopenic obesity (SO), supplement, obesity, insulin resistance, oxidative stress, inflammation, Sea Buckthorn, vitamin D, Omega-3 fatty acids, melatonin, muscle mass, proteins

## Abstract

Obesity causes fat accumulation, and sarcopenia causes loss of muscle mass and strength; together, they worsen insulin resistance and accelerate muscle decline, creating a harmful cycle. Some supplements, along with physical exercise, could be remedies for sarcopenic obesity (SO). In this review, we aim to draw a comparison between supplements studied in experimental research and those evaluated in clinical studies for SO. In experimental studies, Sea Buckthorn—in forms such as oil, freeze-dried powder or pomace—has been shown to enhance muscle cell growth, improve gut microbiota, provide hypoglycemic benefits and increase muscle mass by promoting protein synthesis. Increased consumption of Omega-3 fatty acids may play a protective role against SO in women. Melatonin may positively impact obesity and SO by reducing oxidative stress. Elevated irisin levels, such as those observed with vitamin D supplementation, could prevent muscle wasting and fat gain in SO by improving insulin sensitivity and reducing inflammation. There have been many studies highlighting the potential of vitamin D in preventing age related sarcopenia; however, the effect of vitamin D supplementation in SO is under-researched and appears less promising. Future clinical trials using natural supplements hold promise, as these provide multiple beneficial components that may work synergistically to treat SO.

## 1. Introduction

Obesity is typically associated with caloric excess and fat gain, whereas sarcopenia is marked by the wasting of muscle tissue and progressive loss of skeletal muscle strength [1,2].

Increased intramyocellular lipid content is a common metabolic characteristic of skeletal muscle in aging and obesity. Sarcopenia and obesity exacerbate each other, as muscle loss decreases the amount of insulin-responsive tissue, leading to insulin resistance, which further heightens anabolic resistance [3]. When obesity and sarcopenia overlap, the effects are synergistically detrimental, creating a vicious cycle that exacerbates both conditions [4].

Obesity contributes to a decline in muscle mass and quality, likely due to reduced satellite cell function and impaired mitochondrial dynamics, leading to skeletal muscle loss. Notably, sarcopenia is linked to the atrophy of type II muscle fibers and a decline in satellite cell pools, which are crucial for muscle regeneration [3]. The coexistence of obesity with sarcopenia accelerates muscle mass and function loss [5].

The global prevalence of sarcopenic obesity (SO) in adults is rising rapidly, but the absence of a standardized definition for sarcopenia hinders cross-study comparisons. It is important to know that sarcopenia varies according to race and ethnicity. Thus, small differences in the prevalence of sarcopenia are described as follows: non-Hispanic Whites (11.2–24.3%), Hispanics (21.9–36.0%), non-Hispanic Blacks (4.4–27.7%), and Asians/others (18.5–35.7%) [6]. Inpatients with severe obesity showed a prevalence of 11.48% for SO, which was associated with age (particularly those over 70 years) and gender (higher in women) [7].

## 2. Sarcopenic Obesity (SO)

### 2.1. Risk Factors, Prevalence and Comorbidities for SO

A systematic review and meta-analysis involving 178,546 participants across 21 studies identified risk factors for osteosarcopenic obesity (OSO). The analysis revealed significant associations between OSO and the female gender, physical inactivity, hypertension and frailty. However, no significant associations were found with smoking, alcohol consumption or dyslipidemia [8].

A very recent review, including 20 years of research, underlined the most frequent factors associated with SO, with the top being insulin resistance (50%), dyslipidemia (43%), inflammation (28%), lack of exercise training (30%) and hypertension (27%). Clinical conditions linked with SO development are multiple, but the most frequent are considered aging (100%), diabetes mellitus (20%), osteoporosis (18%), cardiovascular diseases (17%) and postmenopausal status (15%) [9].

SO reduces quality of life and is associated with cardiovascular disease, fractures, dementia and cancer [10,11,12].

The prevalence of metabolic syndrome in relation to SO remains controversial. A recent meta-analysis study based on 12 studies involving 11,308 overweight and obese adults indicated no significant difference in the prevalence of metabolic syndrome between individuals with SO and those without [13].

According to a survey, sarcopenia alone and sarcopenia with obesity were associated with higher mortality risk than obesity alone. However, obesity was linked to a 34% reduced risk of sarcopenia (“obesity paradox”). Sarcopenic obese adults had a 15% lower mortality risk than sarcopenic non-obese adults [14]. Maintaining muscle strength is the key for reducing premature mortality [15].

SO may indicate an earlier stage of biological aging, linked to a greater life expectancy after the age of 65 compared to non-obese adults suffering from sarcopenia [14]. Another meta-analysis showed that SO prevalence in diabetes is 27%, with adverse outcomes including decreased glomerular filtration rate, cognitive decline and insulin resistance, highlighting the need for early screening and intervention [16]. Insulin resistance, a key factor in metabolic dysfunction, exacerbates the progression of SO by impairing muscle protein synthesis and promoting fat accumulation, leading to a vicious cycle of muscle loss and increased fat mass [16].

### 2.2. Insulin Resistance, Inflammation and Oxidative Stress in the Pathogenesis of SO

Certain lipid species, such as ceramides and diacylglycerols, have been identified as inhibitors of the insulin signaling pathway in various models. Their accumulation in skeletal muscle may be attributed to several factors, including a reduced ability of adipose tissue to buffer circulating fatty acids after meals, diminished mitochondrial oxidative capacity or persistent inflammation [17].

Alongside insulin resistance, both inflammation and oxidative stress play key roles in mediating SO. Additionally, muscle fat infiltration (myosteatosis) and mitochondrial dysfunction are involved in the pathogenesis of SO [3].

Inflammation is closely tied to apoptosis, and this is one mechanism that can explain the link between inflammation and SO [18]. Adipose tissue stores fat and regulates interorgan communication, with adipocytes and macrophages producing pro-inflammatory cytokines like IL-6 and TNF-α in response to elevated free fatty acids. In SO, IL-6 and TNF-α are linked to reduced muscle mass and strength, both promoting muscle wasting [3]. There is a causal relationship between multiple inflammatory factors and sarcopenia-related traits. Modern treatment to prevent and address sarcopenia should focus on regulating inflammatory factors [19]. Also, a low dietary inflammatory index was associated with a lower prevalence of sarcopenia [20]. Moreover, by using a multivariable logistic regression model, the researchers demonstrated a positive and significant association between the systemic immune inflammation index and SO [21].

Nowadays, the researchers emphasize that oxidative stress may represent an important pathophysiological link between sarcopenia and obesity. It was demonstrated that oxidative stress increases the catabolic activity and decreases the anabolic pathway in muscle mass [22,23].

### 2.3. Adipokines and Myokines Involved in SO

Leptin resistance, prevalent in obese individuals, impairs normal leptin signaling, reduces fatty acid oxidation in muscles and increases intramuscular fat infiltration, ultimately leading to skeletal muscle dysfunction [24,25,26]. Moreover, prolonged stress, such as high-fat diets, disrupts the protective effect of adiponectin, leaving skeletal muscle vulnerable to metabolic dysfunction [27].

In the same way as adipokines, myokines—including myostatin and irisin—play crucial roles in SO [4]. A positive correlation was observed between fat mass and myostatin levels, while fat mass was negatively correlated with irisin levels [28]. Myostatin binds to the activin type IIB receptor, forming the activin receptor complex with activin-like kinase [29]. This process inhibits myoblast proliferation and differentiation, and it also regulates satellite cell self-renewal while promoting muscle atrophy by suppressing the Akt/mTOR pathway. Elevated myostatin levels are linked to aging, inflammation and SO, where they contribute to muscle loss. Exercise can reduce myostatin levels, making physical activity a crucial intervention for managing SO [30]. Myostatin and Activin receptor type 2 (ActRII) inhibitors have been developed to increase muscle mass and strength, as well as improve insulin sensitivity in vitro. These molecules reduce the expression of myostatin in muscle and fat tissues, making them potentially relevant for treating SO [8]. Irisin, the contradictory myokine to myostatin, is responsible for muscle production and fat reduction. Physical exercise promotes irisin secretion [31].

Moreover, obesity increases the hypogonadism risk, and low testosterone impairs muscle protein synthesis, reduces muscle differentiation and raises fat levels, weakening muscle function and quality [3].

**Figure 1:**
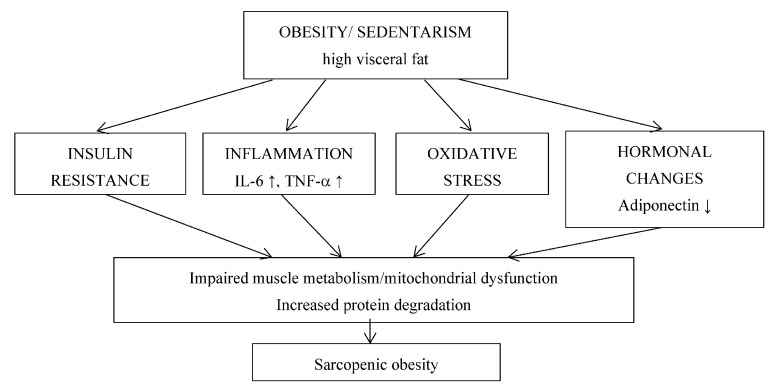
Pathogenic pathways involved in SO.

Insulin resistance, inflammation, oxidative stress and hormonal changes are pathogenic mechanisms in obesity that act synergistically and enhance each other, leading to altered muscle metabolism and SO development [3,4,16,17,18,19,21,22,23,27]. The important pathogenic pathways involved in SO are shown in Figure 1.

## 3. Impact of High-Caloric/High-Fat Diets on Muscle: Experimental Evidence

In 2023, a suitable SO animal model induced by a high-fat diet (HFD) was proposed. Twelve-month-old Sprague–Dawley rats were fed either a high-fat diet (HFD, SO group) or a standard diet (DC group) for 28 weeks, while a younger control group was maintained on a standard diet. Magnetic resonance and histopathological analyses revealed that SO rats (high-fat diet fed) experienced greater muscle loss, strength decline, reduced myofiber number, increased intermyofibrillar mitochondria loss, higher myocyte apoptosis and severe metabolic disorders, including insulin resistance and visceral fat gain. In contrast, DC rats (standard diet-fed) showed only age-related muscle deterioration without significant metabolic disruption compared to the younger control group [32].

By comparing muscle parameters and metabolic indices in obese and non-obese aged rats, Zhu H et al. underlined that the aged obese rats represent the special clinic state of human SO. The findings indicated glycolipid metabolic disorders and insulin resistance in these rats, which were associated with the loss of muscle mass and strength [32].

The high-fat diet (HFD) induces SO in aging female Sprague–Dawley rats by promoting weight gain, metabolic dysfunction, muscle loss and fatty infiltration. HFD disrupts gut microbiota, increases trimethylamine N-oxide (TMAO) levels and impairs the intestinal barrier integrity. TMAO is linked to SO and worsens its progression via the ROS-AKT/mTOR signaling pathway. Thus, the gut microbiota–TMAO–muscle axis plays a key role in HFD-induced SO in aging rats [33].

In another experimental study, male Sprague–Dawley rats, starting at 13 months of age, were fed either a high-fat diet (HFD) or normal diet for 28 weeks to create a rodent model of SO, which was successfully established. RNA-seq analysis of the gastrocnemius muscle in SO rats revealed that differentially expressed genes and alternative splicing events were primarily related to inflammation, immune response, muscle cell and fat cell differentiation and antigen processing. Notably, the transcription factor mef2c (myocyte enhancer Factor 2C) gene, a key regulator of skeletal muscle differentiation and growth, showed significant downregulation and alternative splicing in HFD-induced SO. Genes regulated by mef2c were involved in RNA polymerase II promoter regulation. These findings provide valuable insights into the pathways and mechanisms involved in SO in aging, high-fat-fed rats [34].

Although caloric excess has been shown to have detrimental effects on muscle, it appears that feed restriction is also not beneficial for muscle health. In lambs, feed restriction reduced muscle growth by inhibiting protein synthesis and increasing degradation through the Akt pathway [35]. Anamorelin, an oral ghrelin receptor agonist that promotes lean mass growth, has been suggested to counteract cancer cachexia and is being considered for SO due to its anabolic and anti-inflammatory effects [8].

## 4. Core Treatment Strategies in SO

SO, characterized by the loss of muscle mass and function alongside excess body fat, is a challenging medical condition that significantly impacts quality of life and increases mortality risk. It remains unclear why some individuals with obesity experience muscle decline, despite the fact that anabolic stimuli typically help retain lean mass [7].

Promising treatment approaches focus on targeting the effects of energy overload, including oxidative stress, myosteatosis, inflammation and mitochondrial dysfunction. Various pharmacological treatments for SO include myostatin inhibitors, anamorelin, vitamin D, testosterone and selective androgen receptor modulators. Of course, weight loss and physical exercise are effective approaches to address various pathophysiological factors associated with sarcopenia and obesity [8]. It was shown that physical exercise promotes mitochondrial biogenesis, reduces low-grade inflammation and decreases insulin resistance and skeletal muscle cell apoptosis [36].

Mesenchymal stem cells hold promise as a future therapy for SO due to their immunomodulatory properties and multipotent capabilities [8].

## 5. Supplements for SO Treatment

In this review, we aim to discuss several recommended supplements for the treatment of SO, both in experimental and clinical studies. While it is acknowledged that not all experimental studies can be extrapolated to humans, we have attempted to draw parallels between these findings. Exploring both the successes and limitations of these animal studies can offer insights into which treatments could hold promise and which ones need more rigorous investigation.

Also, in this review, the information is organized starting from the definition of supplements.

A dietary supplement is a product intended for ingestion that, among other requirements, contains a “dietary ingredient” intended to supplement the diet according to the 1994 Dietary Supplement Health and Education Act (DSHEA) Congress. This includes vitamins, minerals, herbs, amino acids, probiotics and related extracts or combinations.

### 5.1. Supplements with Omega-3 Fatty Acids in SO

In an experimental study, male Sprague–Dawley rats were assigned to isoenergetic diets containing 10% fat by weight. A human Western-style diet was mimicked using 5.5% tallow, 2.5% sunflower seed oil rich in n-6 PUFA and 2% olive oil (Control). In the experimental diets, olive oil was replaced with high-DHA tuna oil at low (0.32%) or moderate (1.25%) levels. The fatty acid composition of membrane phospholipids was analyzed in five skeletal muscles chosen to represent a broad range of muscles. As a result of this dietary change, an increase in DHA incorporation into muscle membranes was observed. The experiment demonstrated that Omega-3 fatty acids have beneficial roles in fast oxidative glycolytic muscle fibers [37].

In humans, Omega-3 fatty acids may enhance muscle protein synthesis by activating the mTOR-p70S6K signaling pathway. In older adults, doses of 1650 mg or more per day have been linked to improvements in muscle mass and function, while lower doses showed less benefit. These findings suggest that Omega-3 fatty acids at an appropriate dosage could help combat sarcopenia [38]. Furthermore, higher Omega-3 fatty acid intake has been found to be inversely associated with SO in women, but not in men, according to data from the 2014–2018 Korean National Health and Nutrition Examination Survey. This indicates that an increased consumption of Omega-3 fatty acids may play a protective role against SO in women, highlighting potential gender differences in response to dietary Omega-3 intake [39].

Bird et al. (2021) reviewed 123 studies, and they underlined that Omega-3 long-chain polyunsaturated fatty acids have a positive impact on lean body mass, skeletal muscle mass, handgrip strength and quadriceps muscle strength [40].

### 5.2. Supplements with Sea Buckthorn in SO

The bioactive compounds in Sea Buckthorn, encompassing a diverse array of polyphenols (over 95), fatty acids, vitamins and phytosterols, may play a crucial role in promoting antioxidative and anti-inflammatory effects through their synergistic effects on cellular function and metabolic processes [41]. The polyphenol quercetin, from the Sea Buckthorn fruit and its metabolite isorhamnetin, in low concentrations, was effective in promoting glucose uptake. Hence, the oral administration of quercetin glycoside in mice significantly increased GLUT4 translocation in skeletal muscle. These findings suggest that quercetin and isorhamnetin could be valuable for glucose homeostasis and muscle function, even at physiological concentrations [42].

The hydrophobic branched-chain amino acid peptides derived from Sea Buckthorn seed protein demonstrate significant hypoglycemic effects in type 2 diabetic db/db mice, highlighting their potential to ameliorate insulin resistance and enhance muscle glycogen content through the modulation of key signaling pathways such as PI3K/Akt [43].

Sea Buckthorn oil promotes the proliferation and the myogenic differentiation of sheep primary myoblasts by upregulating proteins like myogenin. It activates the Akt/mTOR pathway, increasing glucose uptake through elevated GLUT4 levels, likely linked to AMPK activation. Additionally, Sea Buckthorn oil inhibits miRNA-292a expression and boosts antioxidative enzymes such as Gpx4 and catalase, indicating its potential as a functional food for muscle growth [44].

Researchers have demonstrated that Sea Buckthorn freeze-dried powder can mitigate obesity and lipid metabolism disorders induced by a high-fat diet. Specifically, administering 4 mg/(g.d. body weight) of Sea Buckthorn freeze-dried powder decreased fat accumulation by downregulating the genes linked to lipid synthesis while upregulating the genes involved in lipolysis. It also improved gut bacteria and impacted important metabolites from microbiota, like acetic acid, propionic acid and butyric acid [45].

In lambs, it was demonstrated that dietary Sea Buckthorn pomace supplementation increased muscle mass and fiber size by promoting protein synthesis via Akt/mTOR signaling and reducing degradation. It improved meat tenderness and HDL content without affecting most serum indices [35].

In humans, the research on supplements containing Sea Buckthorn in SO is relatively limited, indicating a need for further exploration of their potential benefits in addressing health issues.

### 5.3. Supplements with Melatonin in SO

A study on high-fat diet (HFD) mice showed that melatonin and exercise training effectively mitigated sarcopenia in a SO model by preserving satellite cell function and improving mitochondrial health in skeletal muscle [46].

Researchers have revealed an interaction between melatonin and miRNAs, particularly miRNA-483, which may contribute to reduced melatonin secretion in the elderly, playing a role in sarcopenia’s development, highlighting melatonin’s potential as a treatment [47].

Sarcopenia is linked to chronodisruption, particularly to the Bmal1 clock gene. In a study using Bmal1 knockout mice, muscle function, fiber size and mitochondrial capacity were impaired, leading to sarcopenia. Melatonin and exercise proved effective in mitigating these effects, although exercise worsened the mitochondrial damage, suggesting that Bmal1 deficiency contributes to sarcopenia, while melatonin and exercise offer therapeutic potential [48].

### 5.4. Supplements with Vitamins in SO

#### 5.4.1. Vitamin D

The relationship between vitamin D and adipose tissue is intricate. Adipose tissue stores vitamin D and, through vitamin D receptors, influences thermogenesis, inflammation and lipid metabolism [49].

A diet low in protein and vitamin D has been shown to increase the prevalence of obesity, sarcopenia and SO [50]. Sarcopenic patients often develop obesity (sarcopenic obesity) due to the inverse relationship between serum 25(OH)D levels and body fat. Vitamin D deficiency is common in obesity, and since vitamin D inhibits preadipocyte maturation to adipocytes, the low levels of the vitamin reduce this effect, increasing the obesity risk [49].

Maintaining vitamin D levels within the normal range is a key to musculoskeletal health. In 2014, a systematic review and meta-analysis underlined that vitamin D supplementation has a small positive impact on muscle strength [51]. In 2022, Di Filippo et al. emphasized the lack of studies on the effects of vitamin D supplementation in osteosarcopenic obesity and highlighted the necessity for large observational and interventional studies to address this gap [52].

A study that used Mendelian randomization to explore the relationship between genetically predicted 25(OH)D levels and skeletal muscle traits suggested that higher 25(OH)D concentrations are linked to increased grip strength and modest gains in muscle mass, with some evidence of a reduced risk of sarcopenia but not SO [53].

Recent studies have shown that vitamin D supplementation might increase sirtuin 1 and, subsequently, the PPAR-γ coactivator 1α and irisin levels, achieving reduced insulin resistance [54]. Elevated irisin levels, such as those observed with vitamin D supplementation, could prevent muscle wasting and fat gain in SO by improving insulin sensitivity and reducing inflammation. This suggests that interventions targeting irisin may be promising in the prevention and treatment of SO.

Vitamin D may work synergistically with other nutrients like proteins or amino acids to improve musculoskeletal health, offering a potential therapeutic approach for conditions like sarcopenia.

#### 5.4.2. Vitamin K

As an essential nutrient involved in bone metabolism and cardiovascular health, vitamin K is also thought to contribute to muscle function by regulating calcium homeostasis and reducing inflammation, both of which are critical in maintaining muscle mass and strength [55]. Vitamin K has been increasingly linked to muscle health and may play a role in the prevention of sarcopenia.

Low levels of vitamin K have been linked to reduced muscle mass and strength in older adults, indicating that sufficient vitamin K intake might help prevent or slow sarcopenia. While the role of vitamin K supplementation in skeletal muscle health is still largely unexplored, some studies suggest that menaquinone-4 can enhance muscle cell proliferation and improve mitochondrial function. However, the connection between osteocalcin and muscle regulation is being questioned, as recent knockout mouse studies have not confirmed its previously proposed effects on muscle mass and metabolism [56].

### 5.5. Supplements with Proteins in SO

Eglseer et al. studied community dwelling individuals aged 50–70 years with SO who underwent nutritional and exercise interventions for at least 8 weeks. The results demonstrated that resistance training significantly reduced body fat by 1.53% while simultaneously increasing muscle mass by 2.72% and strength by 4.42 kg. Additionally, increasing the protein intake in conjunction with exercise further contributed to a reduction in fat mass [57].

Yang et al. conducted a meta-analysis of randomized controlled trials involving 779 middle-aged and elderly patients with SO. The study found that whole-body electromyostimulation improved muscle mass and reduced waist circumference, while protein supplementation decreased body fat and enhanced grip strength. The combination of whole-body electromyostimulation and protein supplementation yielded additional benefits in skeletal muscle index, grip strength and walking speed; however, there were no significant effects on metabolic or inflammatory biomarkers [58].

## 6. Diet in SO

### 6.1. Antioxidants and Diet

Flavonoids (apigenin, luteolin, quercetin, dihydromyricetin, epigallocatechin gallate and epicatechin) could play a key role in the prevention and treatment of SO by regulating oxidative stress, inflammation, insulin resistance and mitochondrial dysfunction and influencing both anabolic and catabolic processes, as well as satellite cell activity, in skeletal muscle. Recently, a summary table of flavonoids having potential for improving SO was published, but it contained only in vitro and experimental studies, because limited human clinical trials have investigated the effectiveness of flavonoids in SO [59].

Luteolin (found in parsley, celery, thyme, green peppers, chrysanthemum, oregano, artichoke and mint) was investigated in high-fat diet (HFD)-induced obese mice. It was concluded that it effectively reduces obesity, inflammation and protein degradation by inhibiting lipid infiltration into muscle, decreasing inflammatory process and suppressing myostatin and other muscle degradation markers, ultimately leading to improved muscle function [60].

Green cardamom (*Elettaria cardamomum)*, native from Southern India, rich in flavonoids and polyphenols like caffeic acid, gallic acid, quercetin and luteolin, shows potential in treating SO by improving metabolic syndrome factors like diabetes, obesity and hypertension. A study found that 1500 mg daily of green cardamom for 3 months significantly increased irisin, HDLc and insulin sensitivity while reducing fasting insulin, triglycerides, LDLc and fatty liver grade [61]. Thus, green cardamom can prevent metabolic syndrome-related obesity and enhance irisin production, thereby potentially treating SO.

In Table 1, the results from several reviews, meta-analyses and cross-sectional studies from recent years highlight the doses and beneficial effects of supplements such as vitamin D, Omega-3 fatty acids, proteins, melatonin and minerals in SO. Currently, there is a lack of clinical trials testing the medicinal effects of Sea Buckthorn oil, pomace or puree in SO.

### 6.2. Proteins and Diet

Yin et al. conducted a dietary behavior change (a moderate hypocaloric diet with adequate daily protein intake) intervention with 60 community-dwelling older adults (≥60 years) suffering from SO over 15 weeks. The intervention resulted in significant reductions in body weight and improvements in dietary quality. Additionally, participants in the experimental group exhibited enhanced handgrip strength, reduced waist circumference and improved gait speed; however, the muscle mass index decreased. The study noted that cultural factors posed barriers to dietary changes [72].

Perissiou et al. conducted a study on 64 obese adults, comparing the effects of a low-calorie diet combined with exercise to standard dietary advice with exercise over 8 weeks. The results showed that the low-calorie diet improved cardiorespiratory fitness and cardiometabolic profiles but also led to a greater loss of lean muscle mass compared to the standard dietary approach [73].

Hsu KJ et al. studied 69 middle-aged obese adults and found that combining exercise with a high-protein diet over 12 weeks significantly improved muscle power, exercise capacity and physical performance compared to exercise alone [74].

Similarly, Kemmler W et al. examined 100 sarcopenic obese men aged 70 and older over 16 weeks and reported that whole-body electromyostimulation paired with high-protein intake resulted in slight increases in muscle and cardiac biomarkers without impairing renal function [75].

Aparecida et al. (2020) conducted a study involving 111 severely obese participants to evaluate the effects of a DieTBra diet supplemented with extra virgin olive oil over 12 weeks. The results showed significant improvements in walking speed and handgrip strength, along with reductions in total body fat and body weight, with extra virgin olive oil contributing to these positive outcomes [76].

### 6.3. Minerals and Diet

Obese adults are at high risk for deficiencies in vitamins B6, C, D and E; selenium; magnesium and zinc, which may worsen muscle health and sarcopenia. Low-calorie diets add to this risk, making nutrient-dense foods or supplements advisable [77].

In a recent review on the effects of minerals in age-related sarcopenia, only selenium and magnesium were found to be significantly associated with muscle mass, strength, physical performance and sarcopenia risk. No associations were observed for calcium or zinc, and the effects of potassium, iron, sodium and phosphorus on sarcopenia remain uncertain [78].

In SO, selenium and magnesium can be added to the diet to correct existing deficiencies or to achieve higher concentrations that trigger a biological response [3].

Targeted studies on the effects of mineral supplementation in SO have not yet been conducted.

## 7. Conclusions and Future Perspectives

Experimental studies on aged mice fed high-fat diets (HFDs) to model human sarcopenic obesity (SO) have shown promising outcomes with antioxidant and anti-inflammatory compound supplementation. However, such studies are rarely extended to clinical trials in humans. Similarly, while research exists on the benefits of certain phytocompounds that enhance insulin sensitivity and act as antioxidants and anti-inflammatories, as well as micronutrients and trace minerals for obesity or age-related sarcopenia, targeted studies specifically addressing SO remain limited. Currently, SO management focuses on physical exercise and a moderately hypocaloric, high-protein diet, particularly rich in hydrophobic branched-chain amino acids. Supplements such as Omega-3 fatty acids, selenium, magnesium and vitamins D and K are also recommended. Future clinical trials for SO, involving natural supplements like those from Sea Buckthorn fruit, present promising opportunities, as these supplements contain multiple beneficial components that may work synergistically in treating this complex condition.

## Figures and Tables

**Table 1:** Clinical studies on therapeutic doses recommended in SO.

Authors	Study	Sample	Intervention	Result
[62] Moon GK et al. 2023	Review, 21 selected studies from 18,521 studies	Obese subjects	Intake of Omega-3 FA about 2 g/day or intake of proteins, leucine, 250 IU vitamin D3 +Omega-3 FA about 1–2 g/day, for six months	Benefficial effects on muscle health
[63] Therdyothin A et al. 2023	Review on meta analysis and clinical trials	Obese and non-obese subjects	Omega-3 FA > 2 g/day, more than 6 months	Benefits on muscle mass and volume, improved muscle function
[64] Jabbour and Salman 2021	Review, 48 selected studies from 132 studies	7105 morbidity obese subjects (majority females)	Bariatric surgery -follow up the patients 1 week to more than 2 years after surgery	Improved metabolic parameters, muscle strength, physical activity level, etc.
[65] Gray et al. 2013	Review clinical trials	Obese subjects	Omega-3 FA about 2 g/day	Adiponectin ↑, leptin ↑ (prevents weight regain), results considered as indirect muscle benefits
[66] Jabbour et al. 2022	Randomized controled trial	248 overweight older aduts with 25OHvit D baseline (10–30 ng/mL)	Vitamin D supplementation 600 UI vs. 3750 UI/day, >12 months	Higher dose than RDA for vitamin D-no significant muscle improvements
[67] Kawahara et al. 2024	Randomised double blind multicenter trial	1094 subjects with prediabetes 315 overweight and obese prediabetic subjects	Placebo group (*n* = 546) vs. treated group (*n* = 548) with 0.75 µg/day active vitamin D analog for 3 years	Lowering risks of sarcopenia incidence and falls
[68] Espinoza SE et al. 2021	Double blind randomized placebo controlled trials	21 obese, sedentary subjects	Placebo (saline) vs. oxytocin 24 IU in nostrils x4/day, for 8 weeks	Increase in lean body mass
[58] Yang SW et al. 2022	Cross-sectional study using NHANES database in 1999–2002	2532 subjects, average BMI 28 kg/m^2^	Total magnesium intake in milligrams per day calculated from dietary information with 24 h dietary recalls.	Dose dependent relationship between oral intake magnesium and sarcopenia. Sufficient oral intake magnesium might prevent sarcopenia.
[69] Li J et al., 2024	Cross-sectional study	19,696 subjects with BMI 28,6 kg/m^2^	Selenium intake <80.10 μg/day (*n* = 6559), 80.10–124.61 μg/day (*n* = 6569) and >124.61 μg/day (*n* = 6568)	Linear dose response between Se intake and sarcopenia. Se crucial nutrient in reducing risk of sarcopenia.
[70] Lee JY et al., 2014	Cross-sectional study	Seventy-eight Korean postmenopausal women	Relationship between first morning–urine 6-sulfatoxymelatonin (aMT6s) levels (measured as enzyme-linked immunosorbent assay) and sarcopenia (assessed by dual-energy X-ray absorptiometry).	Inverse association between urine melatonin and sarcopenia, suggesting that melatonin may have a protective role in the pathophysiology of sarcopenia
[71] Rondanelli M. et al., 2018	Randomized controled trial	159 elderly sarcopenic patients	Placebo (isocaloric maltodextrin) vs. melatonin 1 mg/daily vs. essential amino acids 4 g/daily vs. essential amino acids 4 g/daily + melatonin 1 mg/daily	Significantly increased fat-free mass in the combined group, treated with aminoacids and melatonin melatonin alone in sarcopenic elderly patients tends to worsen protein metabolism

## References

[1] Donini L.M., Busetto L., Bischoff S.C., Cederholm T., Ballesteros-Pomar M.D., Batsis J.A., Bauer J.M., Boirie Y., Cruz-Jentoft A.J., Dicker D. (2022). Definition and diagnostic criteria for sarcopenic obesity: ESPEN and EASO consensus statement. Obes. Facts.

[2] Cappellari G.G., Guillet C., Poggiogalle E., Pomar M.D.B., Batsis J.A., Boirie Y., Breton I., Frara S., Genton L., Gepner Y. (2023). Sarcopenic obesity research perspectives outlined by the sarcopenic obesity global leadership initiative (SOGLI)—Proceedings from the SOGLI consortium meeting in Rome November 2022. Clin. Nutr..

[3] Axelrod C.L., Dantas W.S., Kirwan J.P. (2023). Sarcopenic obesity: Emerging mechanisms and therapeutic potential. Metabolism.

[4] Polyzos S.A., Margioris A.N. (2018). Sarcopenic obesity. Hormones.

[5] Bilski J., Pierzchalski P., Szczepanik M., Bonior J., Zoladz J.A. (2022). Multifactorial mechanism of sarcopenia and sarcopenic obesity. Role of physical exercise, microbiota and myokines. Cells.

[6] Bigman G., Ryan A.S. (2021). Implications of Race and Ethnicity in Sarcopenia US National Prevalence of Sarcopenia by Muscle Mass, Strength, and Function Indices. Gerontol. Geriatr. Res..

[7] Cancello R., Brenna E., Soranna D., Zambon A., Villa V., Castelnuovo G., Donini L.M., Busetto L., Capodaglio P., Brunani A. (2024). Sarcopenia Prevalence among Hospitalized Patients with Severe Obesity: An Observational Study. J. Clin. Med..

[8] Liu Y., Hao Q., Zhou J., Wu J. (2024). A comprehensive meta-analysis of risk factors associated with osteosarcopenic obesity: A closer look at gender, lifestyle and comorbidities. Osteoporos. Int..

[9] Pinel A., Guillet C., Capel F., Pouget M., De Antonio M., Pereira B., Topinkova E., Eglseer D., Barazzoni R., Cruz-Jentoft A.J. (2024). Identification of factors associated with sarcopenic obesity development: Literature review and expert panel voting. Clin. Nutr..

[10] Wei S., Nguyen T.T., Zhang Y., Ryu D., Gariani K. (2023). Sarcopenic obesity: Epidemiology, pathophysiology, cardiovascular disease, mortality, and management. Front. Endocrinol..

[11] Mirzai S., Carbone S., Batsis J.A., Kritchevsky S.B., Kitzman D.W., Shapiro M.D. (2024). Sarcopenic Obesity and Cardiovascular Disease: An Overlooked but High-Risk Syndrome. Curr. Obes. Rep..

[12] Veronese N., Ragusa F.S., Pegreffi F., Dominguez L.J., Barbagallo M., Zanetti M., Cereda E. (2024). Sarcopenic obesity and health outcomes: An umbrella review of systematic reviews with meta-analysis. J. Cachexia Sarcopenia Muscle.

[13] Khadra D., Itani L., Chebaro Y., Obeid M., Jaber M., Ghanem R., Ayton A., Kreidieh D., EMasri D., Kimura A. (2020). Association between sarcopenic obesity and metabolic syndrome in adults: A systematic review and meta-analysis. Curr. Cardiol. Rev..

[14] Eitmann S., Matrai P., Hegyi P., Balasko M., Eross B., Dorogi K., Petervari E. (2023). Obesity paradox in older sarcopenic adults―A delay in aging: A systematic review and meta-analysis. Ageing Res. Rev..

[15] Sääksjärvi K., Härkänen T., Stenholm S., Schaap L., Lundqvist A., Koskinen S., Borodulin K., Visser M. (2023). Probable sarcopenia, obesity, and risk of all-cause mortality: A pooled analysis of 4,612 participants. Gerontology.

[16] Zhou Y.Y., Wang J.F., Yao Q., Jian Q.F., Luo Z.P. (2023). Prevalence of sarcopenic obesity in patients with diabetes and adverse outcomes: A systematic review and meta-analysis. Clin. Nutr. ESPEN.

[17] Capel F., Pinel A., Walrand S. (2019). Accumulation of intramuscular toxic lipids, a link between fat mass accumulation and sarcopenia. OCL Oilseeds Fats Crops Lipids.

[18] Dirks A.J., Leeuwenburgh C. (2016). Tumor necrosis factor α signaling in skeletal muscle: Effects of age and caloric restriction. J. Nutr. Biochem..

[19] Wang J., Xiang Y., Wu L., Zhang C., Han B., Cheng Y., Tong Y., Yan D., Wang L. (2024). The association between inflammatory cytokines and sarcopenia-related traits: A bi-directional Mendelian randomization study. Eur. J. Clin. Nutr..

[20] Bagheri A., Soltani S., Hashemi R., Heshmat R., Motlagh A.D., Esmaillzadeh A. (2020). Inflammatory potential of the diet and risk of sarcopenia and its components. Nutr. J..

[21] Karanth S.D., Washington C., Cheng T.Y.D., Zhou D., Leeuwenburgh C., Braithwaite D., Zhang D. (2021). Inflammation in relation to sarcopenia and sarcopenic obesity among older adults living with chronic comorbidities: Results from the national health and nutrition examination survey 1999–2006. Nutrients.

[22] Bellanti F., Romano A.D., Buglio A.L., Castriotta V., Guglielmi G., Greco A., Serviddio G., Vendemiale G. (2018). Oxidative stress is increased in sarcopenia and associated with cardiovascular disease risk in sarcopenic obesity. Maturitas.

[23] Gonzalez A., Simon F., Achiardi O., Vilos C., Cabrera D., Cabello-Verrugio C. (2021). The critical role of oxidative stress in sarcopenic obesity. Oxidative Med. Cell. Longev..

[24] Hamrick M.W., McGee-Lawrence M.E., Frechette D.M. (2016). Fatty infiltration of skeletal muscle: Mechanisms and comparisons with bone marrow adiposity. Front. Endocrinol..

[25] Lu W., Feng W., Lai J., Yuan D., Xiao W., Li Y. (2023). Role of adipokines in sarcopenia. Chin. Med. J..

[26] Yang Z.Y., Chen W.L. (2021). Examining the association between serum leptin and sarcopenic obesity. J. Inflamm. Res..

[27] Martinez-Huenchullan S.F., Tam C.S., Ban L.A., Ehrenfeld-Slater P., Mclennan S.V., Twigg S.M. (2020). Skeletal muscle adiponectin induction in obesity and exercise. Metabolism.

[28] Oguz A., Sahin M., Tuzun D., Kurutas E.B., Ulgen C., Bozkus O., Gul K. (2021). Irisin is a predictor of sarcopenic obesity in type 2 diabetes mellitus: A cross-sectional study. Medicine.

[29] Park M.J., Choi K.M. (2023). Interplay of skeletal muscle and adipose tissue: Sarcopenic obesity. Metabolism.

[30] Alizadeh Pahlavani H. (2022). Exercise therapy for people with sarcopenic obesity: Myokines and adipokines as effective actors. Front. Endocrinol..

[31] Kim Y.C., Ki S.W., Kim H., Kang S., Kim H., Go G.W. (2023). Recent advances in nutraceuticals for the treatment of sarcopenic obesity. Nutrients.

[32] Zhu H., Sun Q., Tang H., Chen Y., Tan K., Xu X., Wang S. (2023). A novel rat model of sarcopenic obesity based on aging and high-fat diet consumption. Biogerontology.

[33] Mo X., Cheng R., Shen L., Sun Y., Wang P., Jiang G., Wen L., Li X., Peng X., Liao Y. High-fat diet induces sarcopenic obesity in natural aging rats through the gut–trimethylamine N-oxide–muscle axis. J. Adv. Res..

[34] Sun Q.Q., Zhu H., Tang H.Y., Liu Y.Y., Chen Y.Y., Wang S., Qin Y., Gan H.T., Wang S. (2023). RNA analysis of diet-induced sarcopenic obesity in rats. Arch. Gerontol. Geriatr..

[35] Qin X., Zhang T., Cao Y., Deng B., Zhang J., Zhao J. (2020). Effects of dietary sea buckthorn pomace supplementation on skeletal muscle mass and meat quality in lambs. Meat Sci..

[36] El Assar M., Álvarez-Bustos A., Sosa P., Angulo J., Rodríguez-Mañas L. (2022). Effect of physical activity/exercise on oxidative stress and inflammation in muscle and vascular aging. Int. J. Mol. Sci..

[37] Macartney M.J., Peoples G.E., Treweek T.M., McLennan P.L. (2019). Docosahexaenoic acid varies in rat skeletal muscle membranes according to fibre type and provision of dietary fish oil. Prostaglandins Leukot. Essent. Fat. Acids.

[38] Buoite Stella A., Gortan Cappellari G., Barazzoni R., Zanetti M. (2018). Update on the impact of omega 3 fatty acids on inflammation, insulin resistance and sarcopenia: A review. Int. J. Mol. Sci..

[39] Yang W., Lee J.W., Kim Y., Lee J.H., Kang H.T. (2020). Increased omega-3 fatty acid intake is inversely associated with sarcopenic obesity in women but not in men, based on the 2014–2018 Korean national health and nutrition examination survey. J. Clin. Med..

[40] Bird J.K., Troesch B., Warnke I., Calder P.C. (2021). The effect of long chain omega-3 polyunsaturated fatty acids on muscle mass and function in sarcopenia: A scoping systematic review and meta-analysis. Clin. Nutr. ESPEN.

[41] Chen Y., Cai Y., Wang K., Wang Y. (2023). Bioactive compounds in sea buckthorn and their efficacy in preventing and treating metabolic syndrome. Foods.

[42] Jiang H., Yamashita Y., Nakamura A., Croft K., Ashida H. (2019). Quercetin and its metabolite isorhamnetin promote glucose uptake through different signalling pathways in myotubes. Sci. Rep..

[43] Zhu X., Wang W., Cui C. (2021). Hypoglycemic Effect of Hydrophobic BCAA Peptides Is Associated with Altered PI3K/Akt Protein Expression. J. Agric. Food Chem..

[44] Zhao J., Liang L., Zhang W., Liu X., Huo G., Liu X., Lv X., Zhao J. (2024). Sea buckthorn oil regulates primary myoblasts proliferation and differentiation in vitro. Vitr. Cell. Dev. Biol.-Anim..

[45] Guo C., Han L., Li M., Yu L. (2020). Seabuckthorn (*Hippophaë rhamnoides*) freeze-dried powder protects against high-fat diet-induced obesity, lipid metabolism disorders by modulating the gut microbiota of mice. Nutrients.

[46] Mankhong S., Kim S., Moon S., Lee J.S., Cho E.J., Kwak H.B., Park D.H., Ryu J.K., Kang J.H. (2023). Melatonin and exercise counteract sarcopenic obesity through preservation of satellite cell function. Int. J. Mol. Sci..

[47] Jin H., Xie W., Hu P., Tang K., Wang X., Wu Y., He M., Yu D., Li Y. (2021). The role of melatonin in sarcopenia: Advances and application prospects. Exp. Gerontol..

[48] Fernández-Martínez J., Ramírez-Casas Y., Aranda-Martínez P., López-Rodríguez A., Sayed R.K., Escames G., Acuña-Castroviejo D. (2024). iMS-Bmal1^−/−^ mice show evident signs of sarcopenia that are counteracted by exercise and melatonin therapies. J. Pineal Res..

[49] Lu S., Cao Z.B. (2023). Interplay between Vitamin D and Adipose Tissue: Implications for Adipogenesis and Adipose Tissue Function. Nutrients.

[50] Oh C., Jeon B.H., Storm S.N.R., Jho S., No J.K. (2017). The most effective factors to offset sarcopenia and obesity in the older Korean: Physical activity, Vitamin D and protein intake. Nutrition.

[51] Beaudart C., Buckinx F., Rabenda V., Gillain S., Cavalier E., Slomian J., Petermans J., Reginster J.Y., Bruyère O. (2014). The effects of vitamin D on skeletal muscle strength, muscle mass, and muscle power: A systematic review and meta-analysis of randomized controlled trials. J. Clin. Endocrinol. Metab..

[52] Di Filippo L., De Lorenzo R., Giustina A., Rovere-Querini P., Conte C. (2022). Vitamin D in osteosarcopenic obesity. Nutrients.

[53] Sutherland J.P., Zhou A., Hyppönen E. (2023). Muscle traits, sarcopenia, and sarcopenic obesity: A vitamin D Mendelian randomization study. Nutrients.

[54] Safarpour P., Daneshi-Maskooni M., Vafa M., Nourbakhsh M., Janani L., Maddah M., Amiri F.S., Mohammadi F., Sadeghi H. (2020). Vitamin D supplementation improves SIRT1, Irisin, and glucose indices in overweight or obese type 2 diabetic patients: A double-blind randomized placebo-controlled clinical trial. BMC Fam. Pract..

[55] Aaseth J.O., Finnes T.E., Askim M., Alexander J. (2024). The Importance of Vitamin K and the Combination of Vitamins K and D for Calcium Metabolism and Bone Health: A Review. Nutrients.

[56] Alonso N., Meinitzer A., Fritz-Petrin E., Enko D., Herrmann M. (2023). Role of vitamin K in bone and muscle metabolism. Calcif. Tissue Int..

[57] Eglseer D., Traxler M., Schoufour J.D., Weijs P.J., Voortman T., Boirie Y., Cruz-Jentoft A.J., Reiter L., Bauer S. (2023). Nutritional and exercise interventions in individuals with sarcopenic obesity around retirement age: A systematic review and meta-analysis. Nutr. Rev..

[58] Yang J.M., Luo Y., Zhang J.H., Liu Q.Q., Zhu Q., Ye H., Niu Y.L., Huang H., Xie H.Y., Long Y. (2022). Effects of WB-EMS and protein supplementation on body composition, physical function, metabolism and inflammatory biomarkers in middle-aged and elderly patients with sarcopenic obesity: A meta-analysis of randomized controlled trials. Exp. Gerontol..

[59] Jung U.J. (2023). Sarcopenic obesity: Involvement of oxidative stress and beneficial role of antioxidant flavonoids. Antioxidants.

[60] Kim J.W., Shin S.K., Kwon E.Y. (2023). Luteolin protects against obese sarcopenia in mice with high-fat diet-induced obesity by ameliorating inflammation and protein degradation in muscles. Mol. Nutr. Food Res..

[61] Daneshi-Maskooni M., Keshavarz S.A., Mansouri S., Qorbani M., Alavian S.M., Badri-Fariman M., Jazayeri-Tehrani S.A., Sotoudeh G. (2017). The effects of green cardamom on blood glucose indices, lipids, inflammatory factors, paraxonase-1, sirtuin-1, and irisin in patients with nonalcoholic fatty liver disease and obesity: Study protocol for a randomized controlled trial. Trials.

[62] Moon G.K., Bu S.Y. (2023). Effects of Omega-3 Fatty Acid Supplementation on Skeletal Muscle Mass and Strength in Adults: A Systematic Review. Clin. Nutr. Res..

[63] Therdyothin A., Phiphopthatsanee N., Isanejad M. (2023). The Effect of Omega-3 Fatty Acids on Sarcopenia: Mechanism of Action and Potential Efficacy. Mar. Drugs.

[64] Jabbour G., Salman A. (2021). Bariatric Surgery in Adults with Obesity: The Impact on Performance, Metabolism, and Health Indices. Obes. Surg..

[65] Gray B., Steyn F., Davies P.S., Vitetta L. (2013). Omega-3 fatty acids: A review of the effects on adiponectin and leptin and potential implications for obesity management. Eur. J. Clin. Nutr..

[66] Jabbour J., Rahme M., Mahfoud Z.R., El-Hajj Fuleihan G. (2022). Effect of high dose vitamin D supplementation on indices of sarcopenia and obesity assessed by DXA among older adults: A randomized controlled trial. Endocrine.

[67] Kawahara T., Suzuki G., Mizuno S., Tominaga N., Toda M., Toyama N., Inazu T., Kamahara C., Okada Y., Tanaka Y. (2021). Active vitamin D treatment in the prevention of sarcopenia in adults with prediabetes (DPVD ancillary study): A randomised controlled trial. Lancet Healthy Longev..

[68] Espinoza S.E., Lee J.L., Wang C.P., Ganapathy V., MacCarthy D., Pascucci C., Musi N., Volpi E. (2021). Intranasal Oxytocin Improves Lean Muscle Mass and Lowers LDL Cholesterol in Older Adults with Sarcopenic Obesity: A Pilot Randomized Controlled Trial. J. Am. Med. Dir. Assoc..

[69] Li J., Jiang C., Wu L., Tian J., Zhang B. (2024). Dietary selenium intake and sarcopenia in American adults. Front. Nutr..

[70] Lee J.Y., Kim J.H., Lee D.C. (2014). Urine melatonin levels are inversely associated with sarcopenia in postmenopausal women. Menopause.

[71] Rondanelli M., Peroni G., Gasparri C., Infantino V., Nichetti M., Cuzzoni G., Spadaccini D., Perna S. (2018). Is a combination of melatonin and amino acids useful to sarcopenic elderly patients? A randomized trial. Geriatrics.

[72] Yin Y.H., Liu J.Y.W., Välimäki M. (2023). Dietary behaviour change intervention for managing sarcopenic obesity among community-dwelling older people: A pilot randomised controlled trial. BMC Geriatr..

[73] Perissiou M., Borkoles E., Kobayashi K., Polman R. (2020). The effect of an 8 week prescribed exercise and low-carbohydrate diet on cardiorespiratory fitness, body composition and cardiometabolic risk factors in obese individuals: A randomised controlled trial. Nutrients.

[74] Hsu K.J., Chien K.Y., Tsai S.C., Tsai Y.S., Liao Y.H., Chen J.J., Chen Y.R., Chen C.N. (2021). Effects of exercise alone or in combination with high-protein diet on muscle function, aerobic capacity, and physical function in middle-aged obese adults: A randomized controlled trial. J. Nutr. Health Aging.

[75] Kemmler W., von Stengel S., Kohl M., Rohleder N., Bertsch T., Sieber C.C., Freiberger E., Kob R. (2020). Safety of a combined WB-EMS and high-protein diet intervention in sarcopenic obese elderly men. Clin. Interv. Aging.

[76] Aparecida Silveira E., Danésio de Souza J., dos Santos Rodrigues A.P., Lima R.M., de Souza Cardoso C.K., de Oliveira C. (2020). Effects of extra virgin olive oil (EVOO) and the traditional brazilian diet on sarcopenia in severe obesity: A randomized clinical trial. Nutrients.

[77] Trouwborst I., Verreijen A., Memelink R., Massanet P., Boirie Y., Weijs P., Tieland M. (2018). Exercise and nutrition strategies to counteract sarcopenic obesity. Nutrients.

[78] van Dronkelaar C., Fultinga M., Hummel M., Kruizenga H., Weijs P.J., Tieland M. (2023). Minerals and Sarcopenia in Older Adults: An Updated Systematic Review. J. Am. Med. Dir. Assoc..

